# Management of neglected and fragmented DJ stent with severe encrustation and stone: A case report

**DOI:** 10.1016/j.ijscr.2024.109442

**Published:** 2024-03-11

**Authors:** Ahmed Abebe Mohammed, Sadam Aliye Mohammed, Abeselom Lemma Gebreamlak, Messay Mekonnen Leul

**Affiliations:** a Addis Ababa University College of Health Sciences, Ethiopia; b Addis Ababa University School of Medicine, Ethiopia

**Keywords:** FECal stent, Encrustation, DJ stent, Fragmented

## Abstract

**Introduction and importance:**

DJ stents are widely used in urological procedures and interventions. One of the main problems associated with DJ stent is encrustation and stone formation. The main risk factor for Forgotten, encrusted and calcified (FECal) stent is duration of the stent placement. In addition to high index of suspicion, Imaging like U/S and CT scan are important diagnostic modality. Multiple endourologic and open procedure may be needed for management of fecal stent.

**Case presentation:**

This case report is to discuss a 23 years old female patient with a neglected stent after right pyelolithotomy was done 6 years back. The presence of the stent was identified incidentally after she visited local health facility for recurrent LUTS. The CT scan shows fragmented and encrusted stent with in the bladder and pelvis, stones in stent coils and isolated lower pole stone. She was managed by a procedure of cystolithotomy and right PCNL in separate sessions.

**Clinical discussion:**

Common complication of DJ stent placement especially if left for long duration is encrustation, stent migration, fragmentation and stone formation. Patient or relatives unawareness about the stent placement is the most important cause for neglecting stent. Multiple Endourologic procedures may be needed for the management of FECal stent. However some resource limited settings do combination of endourologic and open surgery.

**Conclusion:**

Minimizing the duration of the stent especially for patient with risk factors is advised to decrease encrustation. Since management of FECal stent is challenging both for patient and urologists, prevention is the way to tackle it. Multiple procedures may be required to manage FECal stent.

## Highlights

• Neglected stent is a rare occurrence but carries huge challenges in urology practices.
• Due to delayed diagnosis and management FECal stents are troublesome and may cause irreversible kidney damage.
• The diagnosis solely relies on detailed patient evaluation and computed tomographic imaging studies.
• Multiple procedures involving both open and endoscopic interventions are needed to manage the condition.

## 1 Introduction

Double-J stents are widely used in our day to day urological procedures and interventions [1]. In any pelvicalyceal procedures and in both diagnostic and therapeutic ureteric procedures they are usually placed to prevent upper tract obstruction be it due to ureteric mucosal edema from manipulations or obstruction due to debris and stone fragments [2]. It is also used as a temporizing drainage measures for sepsis of upper tract blockage due to benign conditions or as a permanent and therapeutic interventions for pelvic malignant conditions [3]. If double-J stents are inadvertently neglected or left in the body for an extended period, they can give rise to serious health complications and pose challenges in subsequent medical management. One of the main problems associated with DJS is encrustation and stone formation on the surface of the stent [4]. For urologists, a retained and forgotten stent syndrome is a syndrome when a stent is forgotten more than two weeks beyond its final limit of stay [5]. The main risk factor for FECal stent is duration. Patients will present with loin pain, repeated treatment for infections, hematuria and dysuria. High index of suspicion and finally imaging with ultrasonography or CT scan of the abdominopelvic is important for diagnosis [6]. Despite we have no strict guidelines in the management of encrusted stents shock wave lithotripsy, ureterorenoscopy and percutaneous interventions are invested [7].

This case is reported on accordance with SCARE criteria [8].

## 2 Case presentation

**Fig. 1:**
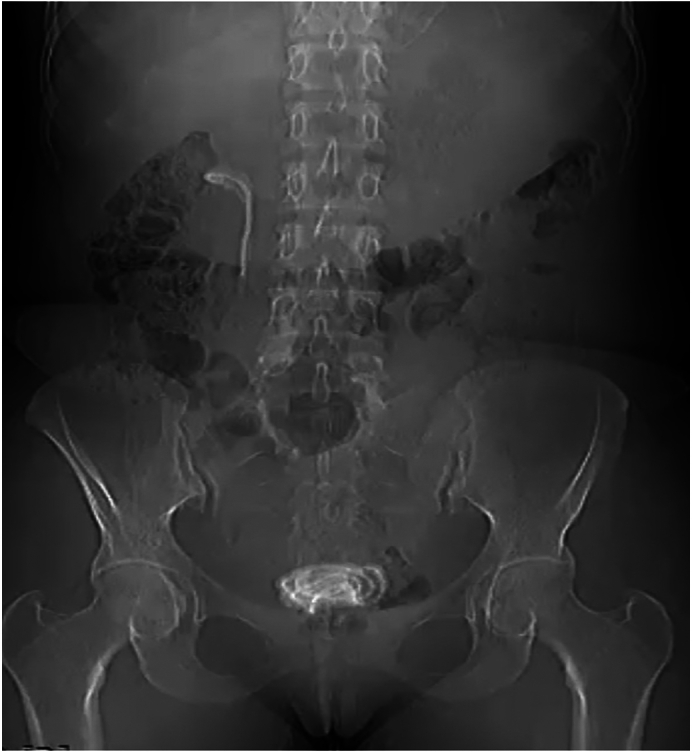
The plain abdominal X ray shows radio opaque shadow both at the bladder and rt. renal shadow area.

**Figs. 2:**
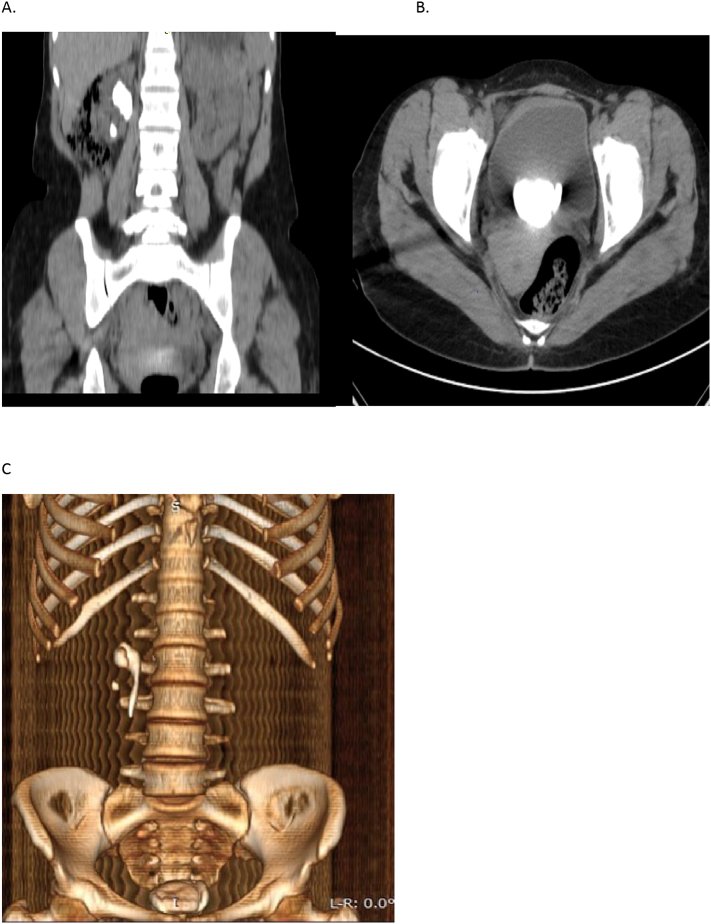
CT scan imaging slices; (A) coronal image showing rt pelvic stone and lower pole stone, (B) an axial image showing huge bladder stone, (C) a 3D reconstruction to show both bladder and rt pelvic stone.

This 23-year-old female has been repeatedly visiting a nearby hospital for worrisome lower urinary tract symptoms in the last two years. For her complaint, she was being treated for a recurrent urinary tract infection. She was not associating the illness with previous procedures. The patient had a history of right pyelolithotomy six years ago for a diagnosis of pelvic stone. There were no remarkable findings on the physical evaluation except for an old surgical scar on the right flank. She was not informed of the placement of the stent during the pyelolithotomy. She lost her follow-up in between. For the above complaints, a plain abdominal X-ray was taken, which showed a radioactively opaque shadow at the pelvic region measuring 45 mm × 27 mm and the right flank area with a size of 12 mm × 75 mm (Fig. 1). During the ultrasonographic imaging of the patient, there was an echogenic material in the bladder with a posterior acoustic shadow measuring 50 mm × 30 mm, and there was a right pelvic echogenic focus with a size of 30 mm and mild pelvicalyceal dilation. A computed tomographic scan was taken, revealing a 51 mm × 13 mm × 30 mm hyperdense area in the bladder. There was also a right pelvic hyperdense area with a size of 70 mm × 20 mm × 30 mm extending to the upper ureter and a 9 mm × 6.8 mm lower pole stone (Fig. 2). Other laboratory investigations were all in normal ranges.

**Fig. 3:**
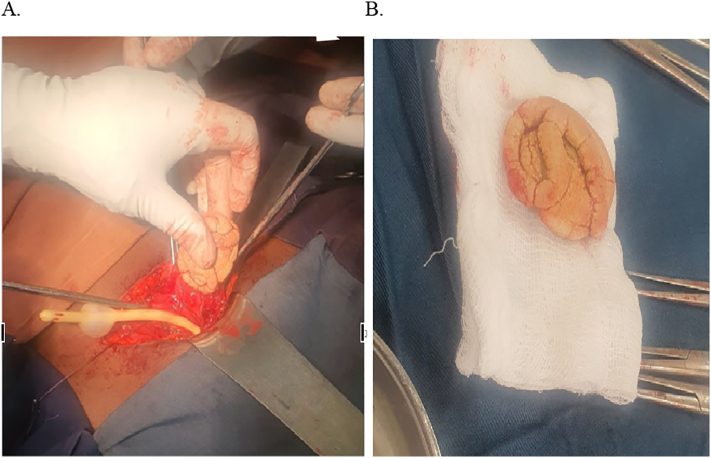
Open Intraoperative finding (cystolithotomy); (A) while extracting the bladder stone with anterior cystotomy, (B) the huge bladder stone on a trolley.

**Fig. 4:**
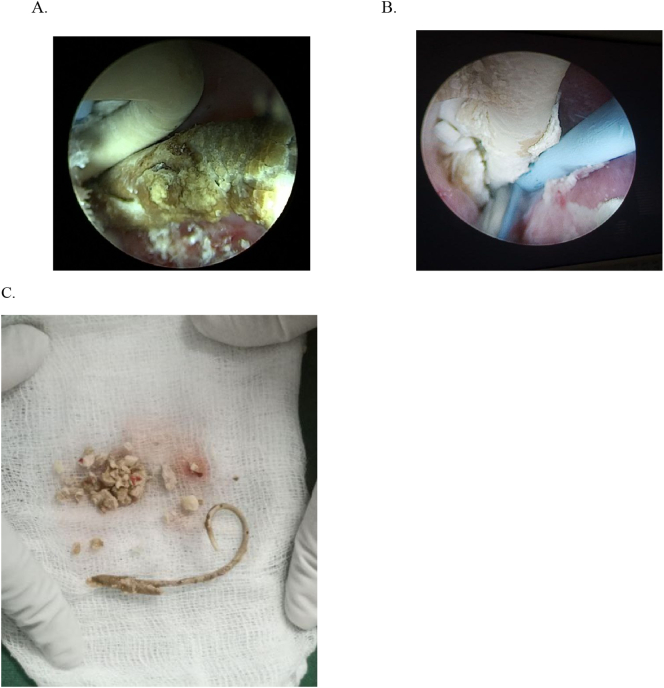
PCNL Intraoperative images: (A) intraoperative finding with encrustation and less encrusted part side by side, (B) hugely encrusted stent and ureteric catheter, (C) fragments after they are removed with the stent.

**Fig. 5:**
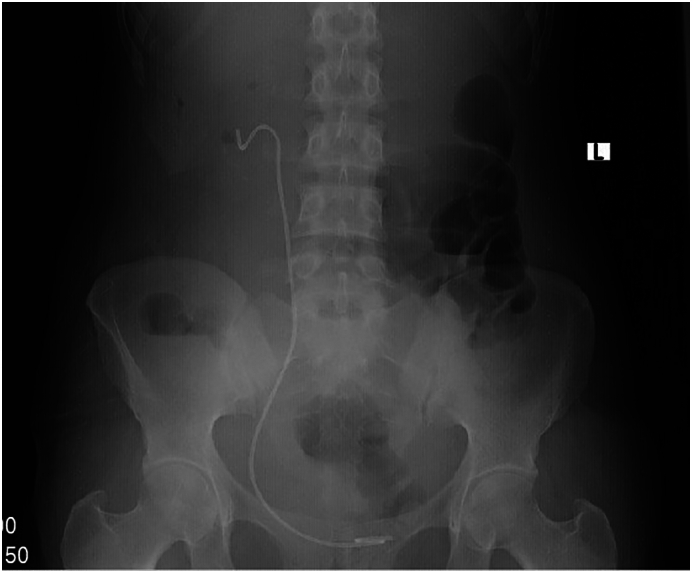
Post-operative control X-ray; it shows a well-placed stent with no residual fragment.

Finally, she was diagnosed with a neglected, encrusted, and fragmented double-J stent with both bladder and right pelvic stones and a lower pole stone. The patient was managed both by open surgery and endourologic treatment in separate sessions. First, the bladder stone is managed via an open cystolithotomy. The intraoperative finding was a stone in the shape of a coiled stent in the bladder. The stone was removed with a 7-cm anterior longitudinal cystotomy (Fig. 3). In the second session, the proximal fragmented stent with the pelvic and lower pole stones was managed with PCNL after 6 weeks of cystolithotomy. The intraoperative finding was an encrusted stent with stone, which is difficult to remove with stone forceps, so pneumatic lithotripsy was used to crush the encrustation and the stone (Fig. 4). The patient is stented with a 6F DJ stent and nephrostomy left in situ. The nephrostomy tube was removed after the control X-ray showed a well-placed DJ stent within 24 h of the PCNL (Fig. 5). Her post-operative course was smooth, and she was discharged after 48 h of surgery. During discharge, the patient was counseled about the presence of the stent and not to neglect it. The stent was removed after 4 weeks of surgery. Her 2-month post-operative ultrasound was unremarkable during follow-up.

## 3 Discussion

Following urologic procedure, ureteral stents are one of the commonly used instrument. DJ stent can be placed prophylactically prior to abdominal or pelvic surgeries for easier identification of ureter, as well as end urological procedure to prevent iatrogenic related complications [9]. After prolonged time stent encrustation is the common complication [10]. To avoid complications, DJ stent should be either removed or exchanged within 6 week to 6 month. If the stent is left for prolonged time serious complication like stent migration, encrustation, fragmentation and stone formation can happen.in our case the stent is not only encrusted and stone formed but also the stent is fragmented in 2 pieces [11]. The most important risk factor for the development of encrustation is duration of stent placement [12]. Other risk factors for encrustation listed in literature include urinary sepsis, chemotherapy, chronic renal failure, metabolic or congenital abnormalities, and nephrolithiasis [10]. It may be common to have minor encrustation on the stent surface, but severe encrustation usually occur in neglected ureteral stent [13]. Because of minerals from urine deposit, the surface of an indwelling stent will encrusted [14]. Although the Causes of neglected stent are multifactorial, patients or relatives unawareness about the stent placement is the most important cause [15]. In one study done in India by Ankur Jhanwar et al. shows that a lack of information (38.16 %), poor economic status (23.32 %), poor adherence of patients of (19.08 %), patients considering it less important to control the inserted stent (12.72 %), and lower educational status (6.36 %) was the factor affecting stent neglection [16].

.In our case patient has different risk factor for FECal stent. The stent was neglected and forgotten for about 6 years because she was unaware of the stent was inserted during her previous stone surgery. The encrustation risk also increases because her previous surgery was stone surgery. She is from low socioeconomic status and low educational level.

To classify severity of stent encrustation; there are two scoring system. These are namely the FECal (forgotten, encrusted, calcified) and KUB (kidney, ureter, bladder) systems [10]. The FECal system classify stent encrustation in to 5 level, being level 1 as minimal encrustation over the coil and level 5 represent encrustation of the whole length of the stent [17].

.Neglected and encrusted DJ stent is a difficult condition for the patient and the managing urologist [18]. For the management of encrusted DJ stent; Combinations of percutaneous nephrolithotomy, shockwave lithotripsy, and ureteroscopy with laser lithotripsy, cystolitholapaxy, and open surgical removal may be needed [19]. In our case patient is managed in 2 sessions, first by open cystolithotomy and second by PCNL with 6 weeks apart.

## 4 Conclusion

Patients usually present with pain and LUTS being totally unaware of stent in them. Duration of the stent is the most determining factor for fecal stent, so minimizing the duration of the stent is important especially in patient with risk factors. Because patient unawareness is the most important risk factor for neglected stent, advising patient should be the part of management during stent insertion. The ways of FECal stent management may require, combination of open and multiple urologic procedure Managing fecal stent is challenging both for the urologist and the patient so, prevention is best way to tackle FECal stent.

## Patient (parent's) consent

Written informed consent was obtained from the patient for publication of this case report and accompanying images. A copy of the written consent is available for review by the Editor-in-Chief of this journal on request.

## Ethical approval

Ethical approval was provided by Ethical review committee of the Department of Surgery, College of Health Sciences, Addis Ababa University.

## Funding

There is no source of funding found for this research paper.

## Author contribution

1. Ahmed Abebe, MD, General Surgeon, and Urology Fellow: conceived, wrote, and submitted the report. Involved in the diagnosis, management and follow up of the patient.
2. Sadam Aliye, MD, Urology resident: Operated on the patient. Involved in the writing of the report and in the follow up of the patient
3. Abeselom Lemma, MD, Assistant professor of urology: Operated on the patient. Reviewed the case report.
4. Messay Mekonnen, MD, Assistant professor, General surgeon and Urologist: Operated on the patient and Reviewed the case report.

## Guarantor

Abeselom Lemma Gebreamlak.

## Research registration number

N/A.

## Conflict of interest statement

All authors declare that they have no conflict of interest.
